# Serotype Features of 17 Suspected Cases of Foodborne Botulism in China 2019–2022 Revealed by a Multiplex Immuno-Endopep-MS Method

**DOI:** 10.3389/fmicb.2022.869874

**Published:** 2022-04-05

**Authors:** Jiang Wang, Hua Xu, Cheng Zhang, Jia Chen, Chunyan Wang, Xinying Li, Yajiao Zhang, Jianwei Xie

**Affiliations:** ^1^Beijing Institute of Pharmacology and Toxicology, Beijing, China; ^2^Yongding Road Outpatient Department, Jingnan Medical District of Chinese PLA General Hospital, Beijing, China; ^3^Poisoning Treatment Department, The Fifth Medical Center of Chinese PLA General Hospital, Beijing, China

**Keywords:** botulinum neurotoxin, foodborne botulism, serotype, endopeptidase-mass spectrometry assay (Endopep-MS), diagnostics, clinical specimens

## Abstract

Diagnosis of botulism caused by multiple serotypes of botulinum neurotoxin (BoNT) is still a challenge due to the lack of a reliable detection method. The present study develops a feasible laboratorial method based on an isotope dilution Immuno-Endopep-MS to detect BoNTs and determine their serotypes and activities in clinical samples. Eleven positive foodborne botulism cases out of a total of 17 suspected cases in China, 2019–2022, were determined by the established method. Blood, urine, vomitus, gastric mucosa samples, and food samples were employed and evidenced to be suitable for the detection. Results showed that, although single type A-intoxication was still the first cause among these foodborne botulism cases, other causes involving type E, type B, and their mixed types were also determined, providing a glimpse to the serotype profile of botulism happened in recent years in China. Furthermore, in order to provide insights into *in vivo* profiles of toxin serotypes, a comprehensive analysis of clinical specimens collected from one family of four patients was performed during a clinically and therapeutically relevant time frame. Serotypes and concentrations of BoNT in specimens revealed a good correlation with symptoms and progresses of disease. Additionally, serum was proved to be more suitable for detection of BoNT/A with a detection window up to 12 days. A urine sample, although rarely reported for foodborne botulism diagnosis, was validated to be suitable for testing BoNTs, with a longer detection window up to 25 days. To the best of our knowledge, this is the first comprehensive analytical research on *in vivo* profiles of serotypes A, B, and E in different types of specimens from mixed botulism cases. Our method and findings facilitate the toxin detection and identification by clinical diagnostic laboratories.

## Introduction

Botulinum neurotoxin (BoNT), a kind of proteolytic toxin produced by certain species of the genus Clostridium, particularly *Clostridium botulinum*, *Clostridium butyricum*, and *Clostridium baratii*, is the most toxic biotoxin known to humankind that causes a paralytic illness called botulism (Schiavo et al., 2000; Arnon et al., 2001; Smith et al., 2015). Foodborne botulism, infant botulism, and wound botulism are the most common poisoning types, which may eventually end in respiratory failure and lead to death without immediate and appropriate treatment (Lonati et al., 2020). In addition, BoNT is well-connected to a large variety of medical applications, and the incidence of iatrogenic botulism is on the rise (Kroumpouzos et al., 2021). Moreover, high toxicity and ease of production make BoNT a likely agent of bioterrorism (Arnon et al., 2001).

Based on antigenic properties, BoNT can be divided into eight serotypes: A to G and FA. Serotypes A, B, E, and, occasionally, F give rise to botulism in humans, C and D are commonly associated with botulism of animals, and type G was usually isolated from soils (Sonnabend et al., 1981; Woudstra et al., 2012; Kroumpouzos et al., 2021). BoNT/FA is a novel serotype discovered in 2016 with a hybrid-like structure of BoNT/A1 and BoNT/F5 (Reynolds et al., 2021). As a 150 kDa protein containing a heavy chain (HC, 100 kDa) and a light chain (LC, 50 kDa) connected by a disulfide bond (Lacy et al., 1998; Maslanka et al., 2016), BoNT has a zinc-dependent metalloprotease activity that cleaves SNARE (soluble N-ethylmaleimide-sensitive factor attachment protein receptor) proteins in peripheral neurons to block the release of neurotransmitters (Swaminathan and Eswaramoorthy, 2000). SNARE is a complex that includes three proteins: VAMP (vesicular-associated membrane protein), SNAP-25 (synaptosomal-associated protein of 25 kDa), and syntaxin. Each BoNT serotype cleaves a SNARE substrate at a distinct cleavage site (Rossetto et al., 2014). BoNT/A, C, and E cleave SNAP-25 atuniquesites of Gln 197-Arg 198, Arg 198-Ala 199, and Arg 180-Ile 181 (Wictome and Shone, 1998; Rosen et al., 2014). BoNT/C also cleaves syntaxin. BoNT/B, D, F, and G cleave VAMP at unique sites of Gln 76-Phe 77, Lys 59-Leu 60, Gln 58-Lys 59, and Ala 81-Ala 82 (Shone et al., 1993; Wang et al., 2015, 2019).

Single BoNT serotype, especially serotype A, is the toxin type most frequently identified; however, mixed toxin production by multiple strains or a single strain of *C. botulinum* may be more common than previously realized (Solomon and Timothy Lilly, 2001; Hansbauer et al., 2016). Serotyping differentiation and identification of BoNT are crucial for diagnosis of botulism, especially for mixed botulism, which is also helpful for epidemiological investigations and source tracing in a timely manner (Thirunavukkarasu et al., 2018). More importantly, the incubation period, symptoms, and treatment are varied with serotypes. The onset symptom of BoNT/E is usually within 24 h, whereas the incubation periods of BoNT/A (0–7 days) and BoNT/B (0–5 days) are much longer (Woodruff et al., 1992; Min et al., 2021) even in some cases up to 10–15 days after ingestion of the contaminated food. Timely administration of serotype-specific antitoxin is an effective way to prevent the progression of neurological syndrome and shorten the duration of supportive treatment at early stage. However, differences in the time of presentation and confused symptoms caused by different serotypes make clinical diagnosis difficult. Rapid and reliable laboratorial detection methods in biological samples are necessary to support clinicians in rapid diagnosis and to help patients receive timely serotype-specific treatment.

To date, various laboratory detection methods have been developed. Mouse bioassay is recognized as the “gold standard” for BoNT detection with a sensitivity of 1 U/ml (approximately 10 pg/ml) (Woodruff et al., 1992; Ferreira, 2001) but with a shortage of time-consuming and usage of live animals. Immunological methods like lateral flow assays (LFAs) are commonly used for the advantages of low cost and easy operation (Notermans and Nagel, 1989). Nonetheless, they are unable to determine the toxin serotype and activity simultaneously (Woodruff et al., 1992). Recently, mass spectrometry-based method has been applied increasingly in toxin detection, owing to its high sensitivity and specificity. The Endopeptidase-mass spectrometry (Endopep-MS) method based on the specific endopeptidase activity of toxin was developed to determine the activity and serotypes of BoNT. Methods for detection of BoNT/A, B, E, and F have been established by using matrix-assisted laser desorption ionization time of flight mass spectrometry (MALDI-TOF-MS) or liquid chromatography tandem mass spectrometry, combined with a multiple reaction monitoring technique [LC-MS/MS(MRM)] (Barr et al., 2005; Ching et al., 2012). Rosen et al. reported a multiplex method for determination of BoNT/A, B, and E in mimic clinical samples, and each BoNT serotype is extracted separately using an individual serotype-specific antibody from the spiked serum samples (Wang et al., 2014). For detection of all seven serotypes, only Boyer reported a MALDI-TOF-MS method using four peptides as seven toxins’ substrates (Boyer et al., 2005). Up to now, there has been no LC-MS/MS-based multiplex method for simultaneous detection of seven serotypes from A to G, and no method has been extensively verified in real clinical samples so that limited information is available about the *in vivo* profile of different serotypes in poisoning individuals.

Herein, a stable isotope dilution LC-MS/MS method for simultaneous detection of BoNT A-G was developed and applied to detect clinical samples. The optimal substrates of each serotype were selected, and their respective stable isotope-labeled internal standards (IS) were employed. Peptide cleavage products and IS peptides of specific serotypes were detected by LC-MS/MS (MRM), which can make a precise differentiation and quantification of seven serotypes. Furthermore, for serotypes A, B and E those cause human botulism an antibody simultaneously specifically recognizing the three serotypes, was used to easily enrich toxins from complex biological matrices. The established method was applied to the analysis of 63 clinical samples from 17 suspected cases in China from 2019 to 2022 to determine the existence of BoNTs and their serotypes. Serum, urine, vomitus, postmortem blood, and gastric mucosa and food samples were comprehensively involved and analyzed. Totally, 11 cases were detected as positive for BoNT. Particularly, a family of four poisoning cases diagnosed as mixed botulism was discussed in detail, and the *in vivo* profile of toxins in biological samples was explored. To our knowledge, this is the first and detailed laboratorial study of BoNT cases by MS technique, which provides insights not only into toxin serotypes causing foodborne botulism in China in recent years but also into the toxico kinetics of toxin serotypes and offers helpful information for early diagnosis and medical treatment of foodborne botulism.

## Materials and Methods

### Materials and Reagents

BoNTs were obtained from the Beijing Institute of Pharmacology and Toxicology (Beijing, China). All peptides were synthesized by Sangon Biotech (Shanghai, China), as shown in Table 1. Monoclonal antibody RE052 specific to BoNT/E and mAb013 simultaneously specific to BoNT/A, B, and E serotypes were kindly provided by Professor Jiannan Feng. Dynabeads^®^Protein G at 30 mg/ml in phosphate buffered saline (PBS) was purchased from Thermo Fisher Scientific (Massachusetts, United States). Ultrapure water (18 MΩ cm^–1^) was generated by a Milli-Q A10 water system Millipore (Boston, MA, United States).

**TABLE 1:** Peptide sequences of substrates, cleavage products, and internal standards for serotypes A–G.

Peptide	Sequence	MRM transition	CE(eV)
AP	Ac-RGSNKPKIDAGNQRATRX^a^LGGR-NH_2_	/	
AP-N	Ac-RGSNKPKIDAGNQ	714.1 > 585.4	37
		714.1 > 261.2	40
AP-C	RATRX^a^LGGR-NH_2_	499.8 > 491.3	29
		333.5 > 288.2	23
ISA	RATRXL(+7)^b^GGR-NH_2_	335.5 > 407.5	21
		335.5 > 288.6	25
BP	LSELDDRADALQAGASQFETSAAKLKRK	/	
BP-N	LSELDDRADALQAGASQ	880.5 > 234.4	40
		880.5 > 433.5	37
BP-C	FETSAAKLKRK	320.6 > 372.2	15
		426.8 > 501.9	21
ISB	LSELDDRADAL(+7)^b^QAGASQ	883.5 > 433.3	33
		883.5 > 234.5	36
CP	Ac-VKYNIDEAQNKASO^c^MGIRRR-NH_2_	/	
CP-N	Ac-VKYNIDEAQNK	682.5 > 611.8	34
		682.5 > 704.4	33
DP	AQVDEVVDIMRVNVDKVLERDQKLSELDDRADALQAGAS	/	
DP-N	AQVDEVVDIMRVNVDKVLERDQK	675.1 > 833.9	25
		540.2 > 625.6	17
DP-C	LSELDDRADALQAGAS	816.2 > 763.8	26
		816.2 > 728.1	33
ISD	LSEL(+7)^b^DDRADALQAGAS	819.6 > 767.5	27
		819.6 > 731.8	29
EP	X^a^GNEIDTQNRQIDRIX^a^EKAD	/	
EP-N	X^a^GNEIDTQNRQIDR	558.3 > 780.2	26
		558.3 > 573.5	23
EP-C	IX^a^EKAD	344.8 > 575.3	14
		344.8 > 227.2	14
ISE	I(+7)^b^X^a^EKAD	348.0 > 462.1	16
		348.0 > 575.1	14
FP	TSNRRLQQTQAQVDEVVDIMRVNVDKVLERDQKLSELDDRADAL	/	
FP-N	TSNRRLQQTQAQVDEVVDIMRVNVDKVLERDQ	757.7 > 660.3	37
		631.6 > 644.0	18
FP-C	KLSELDDRADAL	449.4 > 608.1	13
		449.4 > 572.3	19
ISF	KLSEL(+7)^b^DDRADAL	451.8 > 611.3	13
		451.8 > 575.8	16
GP	Ac-KDELEERAE(hS)^d^LKGAOQFESSAAKLKRRYWWAKL-NH_2_	/	
GP-N	Ac-KDELEERAE(hS)^d^LKGAOQFESSA	599.7 > 622.5	25
		599.7 > 711.9	23
GP-C	AKLKRRYWWAKL-NH_2_	539.9 > 744.5	25
		405.2 > 666.7	19
ISG	AKL(+7)^b^KRRYWWAKL-NH_2_	541.5 > 747.3	24
		541.5 > 536.4	23

*^a^X, norleucine.*

*^b^I (+7)/L (+7): All ^12^C and ^14^N in isoleucine and leucine were replaced with ^13^C and ^15^N, respectively.*

*^c^O, ornithine.*

*^d^hS, homoserine. /no data.*

### Optimization of the LC-MS/MS (MRM) Method

The analysis was performed in a positive mode on a QTRAP^®^ (AB SCIEX, Framingham, MA, United States) 5500 System, combined with an ACQUITY UPLC (Waters, Worcester, MA, United States) in MRM. The column used was a BioBasic™ C18 (Thermo Scientific™, Torrance, CA, United States) column (100 mm × 1 mm, 5 μm). The mobile phase consists of A: H_2_O with 0.1% (vol/vol) formic acid and B: acetonitrile in a linear gradient. A gradient profile was 1% B held at 0–1 min, linearly increased to 25% B over 7 min, and then increased to 95% B over 2 min, and held another 3 min, for a total run time of 12 min. The flow rate was 0.25 ml/min, and the injection volume was 10 μl.

Synthetic product peptides of seven serotypes were used to determine the optimal MRM transitions of the cleavage products of BoNT A-G. Two separate transitions of each peptide were monitored under the optimal collision energy (CE) by LC-MS/MS, as shown in Table 1.

### Quantification of Product Peptides Through Isotopic Dilution

Product peptides were quantified using stable isotope-labeled internal standards. IS of each serotype was consistent with its corresponding NH_2_-terminal (N-terminal) or COOH-terminal (C-terminal) product peptide but was labeled with ^13^C and ^15^N, except the IS of BoNT/C employed the C-terminal product of BoNT/E instead due to the poor chromatographic retention of its own C-terminal product. All the above peptides were dissolved by the ultrapure water. The ratio of analyte peak area relative to the corresponding internal standard (IS) was used to quantify the product peptides, which denoted the amount of BoNT.

The method validation follows the Guidance for Industry Bioanalytical Method Validation of US FDA. The calibration curve, limit of detection (LOD), lower limit of quantification (LLOQ), precision, and recovery of all product peptides were assessed.

### Botulinum Neurotoxin Enrichment and Immuno-Endopep-MS Reaction

A monoclonal antibody, mAb 013, simultaneously specific to BoNT/A, B, and E serotypes, was immobilized on Dynabeads Protein G to enrich toxins from samples. An aliquot of 30 μl antibody-coated magnetic beads was mixed with 1-ml clinical samples by gently rotating for 2 h at RT to capture BoNT. Negative control was serum or urine from healthy donors, while positive control was serum or urine from healthy donors spiked with 2 U corresponding BoNT. Then, the beads were removed and washed three times with a 500-μL PBST buffer (PBS with 0.05% Tween-20), and transferred to another new EP tubes at the last wash. BoNT-captured immunobeads were incubated with a reaction buffer, which contained 50-mM Hepes (pH 7.4), 10-mM dithiothreitol, 10-μM ZnCl_2_, IS of each serotype at different concentrations [ISA = 100 ng/ml, ISB = 2 μg/ml, ISE = 20 ng/ml (ISE is also the IS of BoNT/C), ISD = 1 μg/ml, ISF = 200 ng/ml, ISG = 1 μg/ml], 1 mg/ml bovine serum albumin (BSA), and 0.05-mM peptide substrate mixtures (0.05 mM AP, BP, and EP). All samples were incubated at 37°C for 5 h. The incubation supernatant was collected and added with 5-μL 10% TFA to stop the reaction. The solution was centrifuged at 14,000 × *g* for 5 min prior to LC-MS/MS analysis.

### Clinical Sample Analysis

From July 2019 to January 2022, a total of 63 clinical samples from 17 suspected cases were sent to our laboratory for detection. Of these, 37 samples from 11 cases were tested as BoNT positive, as shown in Table 2. All patients ate industrial foods or homemade foods before the symptom onset and developed botulism-like clinical signs. For example, Patients 1–4 were from one family in Hebei province, a middle-aged man, his parents, and his son together ate vacuum-packed Spanish mackerel, chicken claws, and ham sausage purchased from a local retail store accompanied with homemade pickled Chinese cabbage. Patient 7 was a 53-year-old woman, who ate homemade stinky tofu. After different incubation periods, all patients presented with typical symptoms of botulism, such as nausea, vomiting, fatigue, dysphagia, and so on. Among them, the symptom of Patient 4 was the most severe, who died from respiratory failure within 48 h in a local clinic without antitoxin therapy or mechanical ventilation. While his family members (Patients 1–3) received type A equine botulinum antitoxin (Lanzhou Institution of Biological Products, Lanzhou, China) therapy for 10 days; however, the therapeutic effect was not obvious. They also received trachea intubation and mechanical ventilation for 20 days, 26 days, and 10 days, respectively. LFA strips for BoNT/A were used to preliminarily test the vomitus samples of Patients 1, 2, and 3 on day 1, all showing positive results (Supplementary Figure 1).

**TABLE 2:** Characteristics and detection results of 17 cases of foodborne botulism from 2019 to 2022.

No.	Year	Province	Age	Gender (Relationship)	Incubation period	Symptom	Sample types	Detection results by Immuno-Endopep-MS
1	2019	Hebei	63	Male (Grandfather)	14 h	Nausea, vomiting, abdominal distension, fatigue, dizzy	Day 1: vomitus; Days 3, 6–13: serum; Days 3, 24–25: urine	Positive*
2	2019		65	Female (Grandmother)	14 h			
3	2019		46	Male (Father)	24 h		Day 1: vomitus; Days 3, 6–13: serum; Days 24–25: urine	
4	2019		16	Male (Son)	14 h	Nausea, vomiting, dizzy, respiratory failure	Pericardial blood and gastric mucosa	Positive* (Death case)
5	2019	Inner Mongolia	43	Female	32 h	Vomiting, dysphagia	Day 28: serum, urine	Negative
6	2019	Shanxi	39	Male	18 h	Nausea, vomiting, blepharoptosis,	Day 8: serum	Negative
						dysphagia	Day 8: urine	BoNT/E
7	2020	Xinjiang	53	Female	30 h	Blurred vision, fatigue, dizzy,	Day 11: serum	Negative
						dysphagia(5 days later)	Day 8: urine	BoNT/A
							Stinky tofu	BoNT/A, B
8	2021	Hainan	43	Male	24 h	Nausea, vomiting, dizzy, dysphagia	Day 7: serum	Negative
							Day 7: urine	BoNT/B, E
							Day 7: vomitus	BoNT/A, B, E
9	2021	Hebei	52	Male (Father)	48 h	Dizzy, blurred vision, dysphagia	Day 10: serum	BoNT/A
10	2021		24	Male (Son)	48 h	Dizzy, blurred vision, dysphagia	Day 10: serum	BoNT/A
11	2021		48	Female (Mother)	48 h	Dizzy, blurred vision	Day 10: serum	Negative
12	2021		46	Male (Uncle)	48 h	Nausea, vomiting, dizzy	Day 10: serum	Negative
13	2021	Jiangsu	32	Male (Father)	32 h	Nausea, vomiting	Day 14: Serum, ham sausage	Negative
14	2021		8	Male (Son)	32 h	Nausea, vomiting	Day 14: serum	Negative
15	2022	Henan	48	Male (Friend)	48 h	Vomiting, dizzy, blurred vision	Day 6: serum, urine	Negative
16	2022		55	Male (Husband)	48 h	Vomiting, dizzy, blurred vision	Day 6: serum	Negative
							Day 6: urine	BoNT/A
17	2022		56	Female (Wife)	48 h	Vomiting, dizzy, blurred vision	Day 6: serum, urine	BoNT/A

*Detection results of these positive cases are presented in detail in Table 3.

Fresh clinical samples, including serum, urine, vomitus, succus gastric, postmortem samples, and food samples like stinky tofu, were analyzed upon delivery to our laboratory after collection. A few samples with large volume were aliquoted and preserved at −80°C for further usage, and the frozen samples should be thawed at 4°C before use. All serum, urine, vomitus, and succus gastric samples were centrifuged for 5 min to separate supernatant for detection. Gastric mucosa and stinky tofu were added with 500-μL PBS and 7-mm stainless steel beads to homogenize by a TissueLyser (Schneider Elect, Rueil, France) for 3 min, and, after centrifugation, the supernatant was collected for immuno-enrichment and LC-MS/MS (MRM) analysis.

## Results

### LC-MS/MS (MRM) Method

**FIGURE 1:**
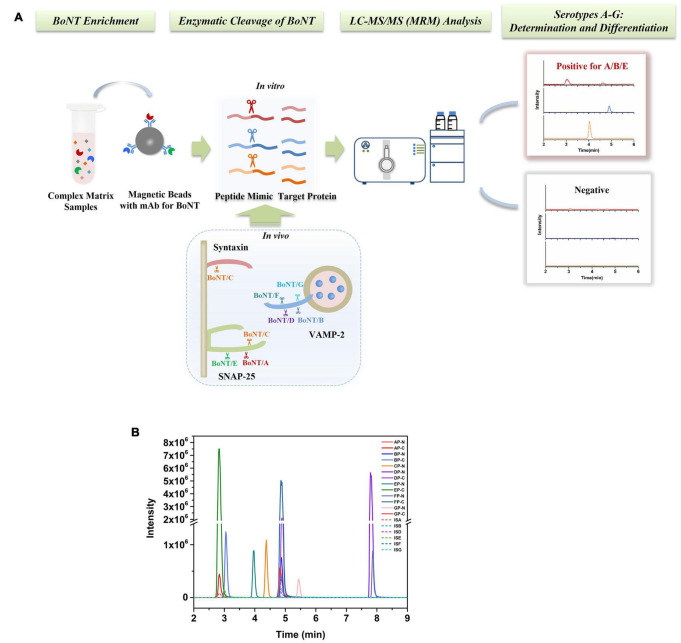
**(A)** Schematic presentation of a stable isotope dilution Endopep-MS method for simultaneous detection of BoNTs A-G. **(B)** LC-MS/MS (MRM) peak intensities for N- and C-terminal cleavage products and their respective internal standard peptides of BoNTs A-G. Synthetic cleavage products of BoNTs A to G presented were at concentrations in the middle of their respective linear ranges.

An Endopep-MS method was constructed for simultaneous detection of BoNTA to G; the method schematic presentation is shown in Figure 1A. Seven peptide substrates, AP-GP, were employed to mimic the targets *in vivo* of BoNTA to G, respectively. Meanwhile, the cleavage products, including N-terminal (such as AP-N) and C-terminal (such as AP-C) peptides of corresponding substrates, were also synthesized, as shown in Table 1. The C-terminal product of BoNT/C has strong polarity, and the retention on reversed-phase columns is poor; therefore, only CP-N (N-terminal) was included in the method. Tandem mass spectrometry was performed by monitoring the precursor to product ion transitions of each peptide under individually optimized conditions. Identification of each product peptide included retention time and a precursor to a product ion multiple reaction monitoring transition match. For example, the doubly charged ion (714.1 *m/z*) of AP-N fragmenting to 585.4 *m/z* and 261.2 *m/z* was monitored, the doubly charged ion (499.8 *m/z*) fragmenting to 491.3 *m/z* and triply charged ion 333.5 *m/z* fragmenting to 288.2 *m/z* of AP-C were monitored. The LC-MS/MS (MRM) peak intensities for N- and C-terminal cleavage products and their respective IS peptides of BoNT A-G are shown in Figure 1B, using synthetic cleavage products of BoNT A to G at medium concentrations of the calibration curve range.

### Performance of the Endopep-MS Method

The LODs and LLOQs of all product peptides were assessed with a standard solution of 10 μg/ml, followed by serial dilution until the signal to noise (S/N) exceeds 5 (LOD) and 10 (LLOQ). The working range starts from the LLOQ to maximize the linearity of the method. At least six concentration levels for each product peptide were consisted in the calibration curves, and it was built by fitting the concentration of the peptides vs. the peak area ratios of the peptide to IS with linear regression (Supplementary Figure 2). The linear regression coefficient *R*^2^ was 0.990–0.999 for all peptides. Results of method validation are shown in Supplementary Table 1. The precision was measured using six replicates, respectively, at low, medium, and high concentrations of peptide standard solutions, resulting in RSD for all samples lower than 15%. The recovery at three different concentrations was within 86–121% (Supplementary Table 2). The concentration and peak area ratios of peptide to IS of FP-N did not exhibit a linear trend; hence, we excluded it in our method.

### Immuno-Endopep-MS Method for a Complex Matrix Sample

**FIGURE 2:**
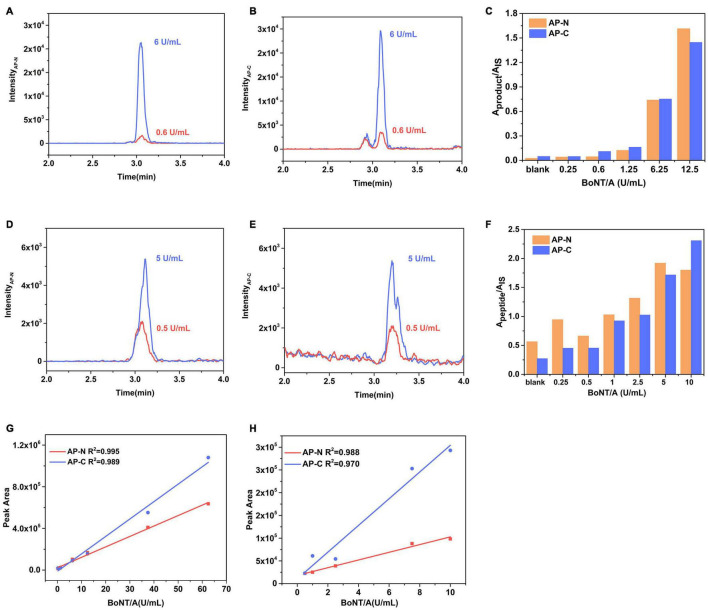
Performance of the Endopep-MS Method in PBS and serum. LC-MS/MS (MRM) peak intensities for **(A)** N-terminal cleavage products (AP-N) obtained at 6 U/ml BoNT/A and LOD (0.6 U/ml BoNT/A), **(B)** C-terminal cleavage products (AP-C) obtained at 6 U/ml BoNT/A and LOD in PBS. **(C)** Comparison of cleavage products of AP from different concentrations of BoNT/A spiked in PBS. LC-MS/MS (MRM) peak intensities for **(D)** AP-N obtained at 5 U/ml BoNT/A and LOD (0.5 U/ml BoNT/A), **(E)** AP-C obtained at 5 U/ml BoNT/A and LOD in serum. **(F)** Comparison of cleavage products of AP from different concentrations of BoNT/A spiked in serum. A calibration curve of BoNT/A spiked in panel **(G)** PBS and panel **(H)** serum.

Human botulism is commonly caused by BoNT/A, B, and E, of which type A exerts the strongest toxicity and causes the most severe symptoms. Here, an Endopep-MS method for clinical sample analysis was constructed using a monoclonal antibody specific to BoNT/A, B, and E. We took BoNT/A as an example for method validation in a complex matrix. Peptide AP, optimized from five different substrates (data not shown), was determined as the substrate of BoNT/A here due to the high enzymatic cleavage efficiency and relatively high mass spectrometry response of its product peptides. The LOD for BoNT/A spiked in PBS was 0.6 U/ml (Figures 2A,B) defined by the toxin, of which the product signal-to-noise ratio (S/N) exceeded three times that of a blank. The signal of AP-C (S/N = 21) at 0.6 U/ml of the toxin exceeded 3 times the signal detected in the blank (PBS) sample (S/N = 4.3). Figure 2C shows the amount of cleavage products AP-N and AP-C under different concentrations of BoNT/A spiked into the Endopep-MS reaction mixture without enrichment. The A_product_/A_IS_ represents the ratio of a peak area of product peptide to the internal standard.

The LOD of the Endopep-LC-MS/MS for BoNT/A was 0.5 U/ml in serum (Figures 2D,E), equivalent to that of the MALDI-TOF MS method using the same substrate, AP (Wang et al., 2013). The amount of cleavage products AP-N and AP-C under different concentrations of BoNT/A is shown in Figure 2F. The linearities for BoNT/A in PBS and serum are shown in Figures 2G,H. As for peptides BP and EP, corresponding to detect BoNT/B and BoNT/E, they have been validated in Rosen’s work (Rosen et al., 2014, 2017); thus, mainly, the LODs were demonstrated here. Their sensitivities under the presence of corresponding BoNT/B and BoNT/E in serum were 1.5 U/ml and 1.8 U/ml (Supplementary Figure 3). Three substrate peptides AP, BP, and EP had no cross reactivity to each other under the presence of corresponding BoNTs.

### BoNT/A, B, and E Detection in Clinical Samples

Totally, 63 clinical samples from 17 suspected cases in China between 2019 and 2022 were sent to our laboratory for confirmation, and 37 samples from 11 cases were detected positive for BoNT. Serum, urine, vomitus, postmortem samples, and food samples were employed. For 11 positive cases, there were 4 cases (Patients 9, 10, 16, and 17, accounted for 36%) determined as type A, 3 cases (Patients 2, 3, and 4, 27%) were type A/E, 2 cases (Patients 1 and 8, 18%) were type A/B/E, 1 case (Patient 6) was type E, and 1 case (Patient 7) was type A/B. Especially, a series of samples on different onset days collected from a family of Patients 1–4 were determined.

#### Serum Samples

**FIGURE 3:**
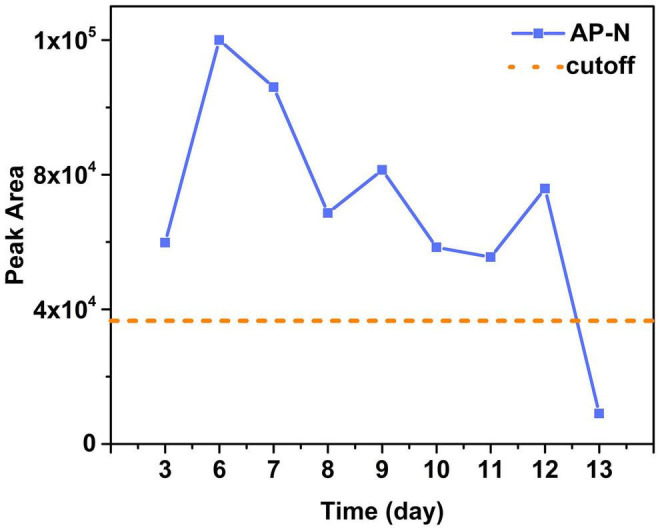
The peak areas of AP-N in sera of Patient 2 on different days and the cutoff value (three times of the blank). Results showed sera of Patient 2 were positive for BoNT/A on day 3 to day 12 with a maximum on day 6 and then gradually decreased.

**FIGURE 4:**
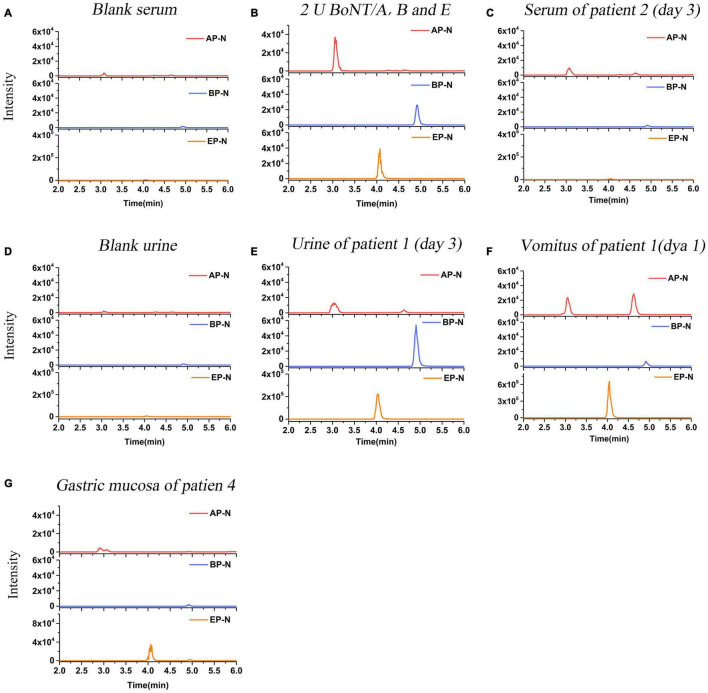
LC-MS/MS (MRM) peak intensities for N-terminal products of BoNT/A, B, and E in clinical samples. **(A)** N-terminal products obtained in blank serum. **(B)** N-terminal products obtained in serum spiked with 2 U BoNT/A, B, and E (positive control). **(C)** N-terminal products obtained in serum samples of Patient 2 on day 3 after the symptom onset. The results showed serum of Patient 2 was positive for BoNT/A, and negative for BoNT/B and E. **(D)** N-terminal products obtained in blank urine. **(E)** N-terminal products obtained in urine samples of Patient 1 on day 3. The results showed urine was positive for BoNT/A, B, and E simultaneously. **(F)** N-terminal products obtained in the vomitus sample of Patient 1 on day 1. The results showed the vomitus sample was positive for BoNT/A, B, and E simultaneously. **(G)** N-terminal products obtained in the gastric mucosa sample of Patient 4. The results showed the sample was positive for BoNT/E, and negative for BoNT/A and B.

Serum samples of Patients 1–17 (except Patient 4) were collected and detected. Among them, serum samples of Patients 1–3, 9–10, and Patient 17 were positive for BoNT/A, as shown in Table 2. For the family, serum samples of Patients 1–3 collected on day 3 after the symptom onset; only Patient 2 was detected positive for BoNT/A (Table 3). While, on day 6, all sera of these three patients showed positive for BoNT/A; meanwhile, Patient 2 also showed positive for BoNT/E. The concentration of BoNT/A in the serial serum samples of individual patients showed a highest level on day 6. For example, the concentration of BoNT/A was maximum on day 6 in the sera of Patient 2 and decreased gradually from day 6 to day 12, as shown in Figure 3. Chromatogram of cleavage products in blank serum, positive control (2 U BoNT/A, B, and E in serum), and a serum sample of Patient 2 collected on day 3 is shown in Figures 4A–C.

**TABLE 3:** BoNT/A, B, and E detection results in serum, urine, vomitus, and anatomical samples of Patients 1–4.

Onset days	BoNT serotypes	Serum			Urine			Vomitus			Pericardial blood	Gastric mucosa
			
		P1	P2	P3	P1	P2	P3	P1	P2	P3	P4	
1	A	/			/			++	−	−	/	
	B							+++	−	−		
	E							+++	+	+		
3	A	−	+	−	+	+	/	/			+*	−
	B	−	−	−	+	−					−	−
	E	−	−	−	+++	+					−	++
6	A	++	++	++	/			/			/	
	B	−	−	−								
	E	−	+	−								
7	A	−	++	−	/			/			/	
8	A	±	±	±	/			/			/	
9	A	+	+	+	/			/			/	
10	A	−	+	−	/			/			/	
11	A	±*	±*	−	/			/			/	
12	A	−	+	−	/			/			/	
13	A	−	−	−	/			/			/	
24	A	/			+	+	−	/			/	
	B				−	−	−					
	E				+	−	−					
25	A	/			+	+	−	/			/	
	B				−	−	−					
	E				−	−	+					

P1, Patient 1; P2, Patient 2; P3, Patient 3; P4, Patient 4. +, peak areas of cleavage products exceed three to ten times the product of a blank. ++, cleavage products exceed ten to thirty times the product of a blank. +++, cleavage products exceed thirty times the product of a blank. −, negative result. /, no sample, no data. ±*, a hemolytic sample. The peak areas for AP-N and AP-C in hemolytic samples were near to the cut off value (three times of the blank).

#### Urine Samples

Urine samples of Patients 1–3, 5–8, and Patients 15–17 were collected. Patients 7, 16, and 17 were positive for BoNT/A, while Patients 6 was positive for BoNT/E; Patient 8 was also shown positive for BoNT/B and E (Table 2). Chromatogram of cleavage products in a blank urine sample is shown in Figure 4D. For Patient 1, the urine was positive for BoNT/A, B, and E (Figure 4E) on day 3, still positive for both BoNT/A and E on day 24, and only positive for BoNT/A on day 25. Contrast to the relative high level of toxins in the urine sample on day 3, BoNT/B and E were negative on day 25. For Patient 2, the urine taken on day 3 was positive for both BoNT/A and E and only positive for BoNT/A on days 24–25. For Patient 3, the urine of day 25 was positive for BoNT/E (Table 3).

#### Vomitus Samples

The vomitus samples were only collected from Patients 1–3 on day 1 and Patient 8. For Patient 1, it was positive for BoNT/A, B, and E simultaneously, as shown in Figure 4F. For Patients 2 and 3, it was only positive for BoNT/E (Table 3). It was worth noting that the detected BoNT/E level of Patient 1 was higher than those of Patients 2 and 3, which was about 20–30 times of the latter. The vomitus sample of Patient 8 was positive for BoNT/A, B, and E simultaneously. LFA was also used to test the samples from Patients 1–3 with a BoNT/A positive result.

#### Postmortem Samples

Pericardial blood and gastric mucosa were collected from the deceased individual (Patient 4). The pericardial blood sample was positive for BoNT/A despite hemolysis. The gastric mucosa was positive for BoNT/E (Table 3). Figure 4G shows the N-terminal cleavage product of BoNT/E.

#### Food Samples

Due to the limited access of suspected food samples, we collected remained stinky tofu samples consumed by Patient 7 and ham sausage consumed by Patient 13 for detection, which showed Patient 7 positive for both BoN/A and B.

## Discussion

Botulism is a life-threatening disease in humans and animals, which is caused by active BoNT. Timely diagnosis and treatment of botulism are crucial for survival due to the extreme potency and short onset time like E serotype (Woodruff et al., 1992), which relies on the laboratory confirmation. In addition, epidemiological investigation after an outbreak and source tracing also relies on the establishment of the detection method (Thirunavukkarasu et al., 2018). In China, conventional laboratory procedures for botulism confirmation are bacterial culturing, LFA, and mouse bioassay (Dong et al., 2017; Xin et al., 2019). However, bacteria isolation and culturing detect *C. botulinum* rather than the active toxin; thus it is not sufficient to confirm the clinical diagnosis, and the whole procedure takes at least 7 days (Xin et al., 2019). Mouse bioassay can detect active BoNT in contrast to PCR assay; however, it usually takes 1–4 days to complete and needs live animals. Here, a convenient multiplex Endopep-MS assay was established, which can make an activity determination and serotype differentiation of BoNT A-G.

In our method, seven synthetic peptides, i.e., AP-GP, were package used to imitate the substrates *in vivo* of BoNT A-G. Substrate AP has been applied to Endopep-MS assay for BoNT detection by MALDI-TOF MS (Wang et al., 2013), but it has not been used by LC-MS/MS (MRM). BP and EP have been validated both in a single and multiplex Endopep-MS assay (Rosen et al., 2014, 2015). CP and GP were the optimized peptide substrates in Wang’s work for BoNT/C and G, respectively, which were validated in the MALDI-TOF MS method (Wang et al., 2015, 2019). Here, product peptides of BoNT A-G were quantified using the stable isotope dilution LC-MS/MS method, of which the validation results met the methodological requirements. The narrow linear range and relatively poor mass response of the N-terminal product peptides of serotypes B, D, F, and G are probably due to the large molecular weight and more acidic amino acids, while their respective C-terminal peptide performs well. Presence of a product peptide at either N- or C-terminal in a sample was recognized as a positive result for BoNT.

Human botulism is commonly caused by BoNT/A, B, and E, and, recently, mixed serotypes have occurred increasingly worldwide, so we further focused on the establishment of a method to simultaneously detect BoNT/A, B, and E in biological matrix. The clinical samples commonly used for diagnosis of botulism include serum, stool, or gastric contents (mainly for foodborne botulism). The complexity of these samples poses a challenge to the BoNT detection. Here, we employed the BoNT-specific antibody-coated beads to extract BoNTs from clinical samples. In Rosen’s work, serotypes A, B, and E were extracted separately using the mono-serotype-specific antibodies (Rosen et al., 2017). In our work, the usage of antibody mAb013, a monoclonal antibody simultaneously specific to BoNT/A, B, and E, can simplify the enrichment procedure and thus shorten the analysis time. Under the optimized immunocapture multiplex Endopep-MS method, the sensitivity for BoNT/A was 0.6 U/ml in PBS and 0.5 U/ml in spiked serum. The sensitivity of our method was in the same order of magnitude as reported by Rosen et al. (2017). LODs of BoNT/B and E in spiked serum were 1.5 U/ml and 1.8 U/ml, respectively; both exhibited a good sensitivity and specificity.

Importantly, the method was successfully used to detect clinical samples from suspected patients, and the whole analysis process only took an average of 7.5 h for various clinical samples. We performed a retrospective summary of the samples tested in the past 3 years. Overall, 37 out of 54 samples of 11 cases were determined BoNT positive out of 63 clinical samples from 17 suspected cases. Reasons for these BoNT-negative samples are possibly due to the following aspects: time of sampling far beyond the detection window, trace amounts of ingested BoNT, individual differences among patients, and samples collected from non-botulism cases, although exhibited similar symptoms. The median age of patients was 43 years (range: 8–65 years); 12 were male. Among the positive 11 cases, the median age was 46 years; 8 were male. The detection results were consistent with the symptoms presented by the patients and provided useful information for physicians to improve the treatment procedures. For the positive samples, botulinum neurotoxins type A (4 cases, 36%) was the toxin type most frequently identified, followed by toxin type A/E (3 cases, 27%), toxin type A/B/E (2 cases, 18%), toxin type A/B (1 case), and toxin type E (1 case). Results demonstrated that mixed botulism cases may be more common than previously realized. This phenomenon is possibly due to the lack of multiple detection methods for different serotypes in the past years. Changes of the distribution and epidemiology of different serotypes maybe caused by fast development of logistics delivery would also contribute to the increase of mixed botulism. The results demonstrate the necessity of development a multiplex laboratorial diagnosis method for BoNTs. Different kinds of samples are evidenced to be suitable for detection *via* the established Immuno-Endopep-MS method, revealing a broad applicability of our method.

Despite reports of poisoning cases involving multiple serotypes, there is no report on the detailed *in vivo* profiles of toxins involving serotypes, concentrations, detection window, and preferred distribution. In order to take a look at the *in vivo* profiles of toxins in poisoning patients, a family involving Patients 1–4 is discussed in detail as follows, and the timeline on these mixed botulism cases is presented (Supplementary Figure 4 and Table 3).

For Patient 1, the urine sample taken on day 3 and the vomitus sample taken on day 1 were both positive for BoNT/A, B, and E. In these two samples, the level of BoNT/E was the highest among the three serotypes, and it was more than 10 times higher in Patient 1 than in Patients 2 and 3. The concentration of BoNT/A in urine was about three times higher than that for Patient 2, and the urine samples taken on days 24 and 25 were also positive for BoNT/A. Meanwhile, BoNT/B was only detected in the urine sample of Patient 1. Symptoms and the period of ventilation of Patient 1 were more severe and longer than Patient 3, which was consistent with our detection results that both concentrations of BoNT/A and E of Patient 1 were higher than those of Patient 3. For Patient 2, serum samples were positive for BoNT/A on days 6–12, with a wider detection window vs. Patient 1 and Patient 3 (Day 9), and the level of BoNT/A in serum on day 6 was also the highest among three patients. The vomitus sample collected on day 1 was only positive for BoNT/E. The urine sample showed a positive result in BoNT/A and E on day 3, and it was still positive for BoNT/A on day 25. The duration of mechanical ventilation for Patient 2 was the longest among the three patients due to her being uncooperative with treatment and poor health condition. Unfortunately, Patient 2 deceased 2 months after the discharge, mainly due to high blood pressure combined with diabetes and other diseases, and may also be due to the incomplete removal of BoNT from the body. For Patient 3, the serum sample was positive for BoNT/A on day 6 and turned to negative on day 10. The vomitus sample was only positive for BoNT/E, and the urine sample taken on day 25 was positive for BoNT/E. Symptoms of Patient 3 were the mildest, and his recovery was the fastest, which was consistent with the result that the level of BoNT/A in serum was the lowest among the three patients. The fast recovery may also due to his strong physical condition. For Patient 4, results indicated that BoNT/A was positive in serum, and BoNT/E was detected in gastric mucosa samples. The food eaten by Patient 4 was far more than that taken by Patients 2 and 3; therefore, it is more likely that the amount of BoNT ingested by Patient 4 was more than the other patients. As a result, Patient 4 presented the most significant symptoms and unfortunately ended in respiratory failure.

Four categories of clinical samples from the four patients were analyzed using our method. We observed that all serum samples were positive only for BoNT/A except one sample of Patient 2 on day 6 was also positive for BoNT/E. Our observation coincided with the finding of Woodruff et al. that samples from patients with type A botulism tend to be positive in serum than those from patients with type B or E botulism (Woodruff et al., 1992). Results also indicated that serum is especially suitable as a sample for BoNT/A botulism confirmation with a detection window up to 12 days (Figure 3) but may not be so suitable for other serotypes. The vomitus sample is a valuable sample during the early stage of foodborne intoxication, which provides an original source reflecting the BoNT ingestion. A urine sample is rarely reported to be used in laboratory confirmatory assays for botulism; however, our results indicated that a urine sample is suitable for testing multiple serotypes of BoNT and may provide a longer detection window up to 25 days. These findings provide important data for laboratorial investigation of BoNT poisoning.

The onset time of above four patients was about 14 h after the dinner, which was consistent with the feature that BoNT/E has the shortest incubation period among serotypes A, B, and E. BoNT/E exerts toxicity more quickly than serotypes A and B due to its fast speed to enter neurons and to block the neurotransmission (Jiafu et al., 2008). The fatality rate in patients with type E foodborne botulism was reduced after receiving appropriate antitoxin treatment (Mottate et al., 2016). Therefore, individuals who were exposed to BoNT/E should be admitted to hospital and receive antitoxin therapy as early as 24 h after ingestion of contaminated food. Patient 4 with the most significant symptoms was misdiagnosed as acute gastroenteritis in the local clinic, hence missing the optimal treatment window.

The main symptoms of Patients 1–4 were autonomic dysfunction like nausea, vomiting, dizziness, and fatigue, which were in accordance with the characteristics of BoNT/E intoxication (Woodruff et al., 1992). Botulism from BoNT/A generally results in more severe disease presented as bulbar nerve dysfunction and skeletal muscle impairment, such as dysphagia, diplopia, and dyspnea, which usually require mechanical ventilation up to several months (Hughes et al., 1981; Hellmich et al., 2018). In our case, Patients 1–3 all required relative short ventilation support treatment for 10–30 days. It is worth noting that Patients 1–4 did not present dysphagia and blurred vision, which occurred significantly more frequently in patients with type A botulism (Hughes et al., 1981) just like Patients 7, 9, and 10 shown in Table 2. The non-obvious symptoms implied that Patients 1–4 were not mainly intoxicated by BoNT/A. Combining with the experimental results of Endopep-MS and the fact that recovery from serotype E is faster than serotype A (Eleopra et al., 1998), we considered the patients were intoxicated mainly by serotype E, accompanied with serotype A or both A and B.

In order to further validate the serotype E-dominated results, E serotype-specific mAb RE052 was used to capture the vomitus and urine samples in which BoNT/E showed positive when captured by mAb013. The results were consistent with those of mAb013. It also made an explanation for the non-obvious therapeutic effect of Patients 1–3 receiving type A equine botulinum antitoxin after detecting BoNT/A by LFA (Supplementary Figure 1). In addition, vomitus samples were cultured for *C. botulinum*, which only showed serotype E-positive. Together, it demonstrated that LFA cannot accurately differentiate the serotypes of BoNT, while culturing may be more prone to identify the dominant growing strains and overlook some non-dominant strains, in turn highlighting the specificity and efficiency of the multiplex immuno-Endopep-MS method, especially in the diagnosis of mixed botulism.

Mixed botulism may be caused by different individual strains, producing different types of toxins, or by certain rare strain, which is known to produce more than one serotype. For example, BoNT/A2, F4, and F5 were produced by a *C. botulinum* strain named Af84 (Kalb et al., 2014). In a case of infant botulism, *C. botulinum* type Af has been isolated and cultured from the stool sample (De Jong et al., 2015). However, here, specimens were handled without further anaerobic isolation and culturing except vomitus samples; therefore, we do not know the exact type of the pathogenic strains and whether existing a multi-toxin-producing *C. botulinum* strain in these mixed serotypes validated clinical samples.

## Conclusion

We developed a stable isotope dilution Endopep-MS method for serotype differentiation and activity determination of BoNTs A-G, which is validated to meet the bioanalytical requirements. A method for serotypes A, B, and E detection in a complex matrix using single antibody-coated magnetic beads was further constructed and successfully applied to five types of clinical samples. During 2019–2022, 11 positive foodborne botulism cases have been confirmed in our laboratory whose detection results were consistent with the clinical symptoms and progresses of disease. The need to monitor multiple toxin intoxication has been demonstrated due to the mixed botulism cases that were evidenced to be more common in China in recent years, and the characteristics of intoxication by different serotypes reveal disease trends, which is meaningful for the clinical treatment. A family involving four cases of mixed botulism with predominantly type E toxin was further discussed in a clinically and therapeutically relevant time frame to explore the *in vivo* profile of toxins. The established multiplex Endopep-MS method with high sensitivity and specificity was proved to be of great significance for timely and accurate diagnosis of botulism even a trace to the source during an outbreak. It can be a toxicological alternative or complementary to traditional surveillance detection and would be useful to elucidate how toxins distribute and circulate *in vivo* and offers a feasible way to clinical diagnosis of mixed botulism.

## Data Availability Statement

The original contributions presented in the study are included in the article/Supplementary Material, further inquiries can be directed to the corresponding authors.

## Ethics Statement

The study was reviewed and approved by the Ethics Committee of Chinese PLA General Hospital. The patients/participants provided their written informed consent to participate in this study.

## Author Contributions

HX and JX contributed to the idea and design of this research. JW performed the experiments and wrote the manuscript. HX designed the experiments and revised the manuscript. JC and YZ were involved in the data analysis. CZ, CW, and XL contributed to the resources of this research. All authors have read and approved the final manuscript.

## Conflict of Interest

The authors declare that the research was conducted in the absence of any commercial or financial relationships that could be construed as a potential conflict of interest.

## Publisher’s Note

All claims expressed in this article are solely those of the authors and do not necessarily represent those of their affiliated organizations, or those of the publisher, the editors and the reviewers. Any product that may be evaluated in this article, or claim that may be made by its manufacturer, is not guaranteed or endorsed by the publisher.
