# Supplementary material for: Serotype Features of 17 Suspected Cases of Foodborne Botulism in China 2019–2022 Revealed by a Multiplex Immuno-Endopep-MS Method
Source: Front Microbiol. 2022 Apr 5;13:869874. doi: 10.3389/fmicb.2022.869874 (PMC9016322; doi:10.3389/fmicb.2022.869874)
Supplement: Supplementary file 1 [file Data_Sheet_1.docx]

Supplementary Material

## Supplementary Tables

**Table S1** Linearity, working range, LOD and LLOQ of product peptides

| Peptide | Working Range  (ng/mL) | LOD  (ng/mL) | LLOQ  (ng/mL) | Linearity  Regression equation | R^2^ |
| --- | --- | --- | --- | --- | --- |
| AP-N | 1.25-1000 | 0.15 | 1.25 | y = 8.42E-04x + 5.41E-02 | 0.992 |
| AP-C | 6.25-1000 | 3.12 | 6.25 | y = 5.80E-03x – 4.91E-02 | 0.998 |
| BP-N | 250-2000 | 250 | 250 | y = 1.44E-04x – 5.78E-03 | 0.999 |
| BP-C | 25-1000 | 0.62 | 25 | y = 3.90E-03x + 5.15E-01 | 0.996 |
| CP-N | 1.25-1000 | 0.62 | 1.25 | y = 1.76E-02x – 2.91E-01 | 0.996 |
| DP-N | 2000-10000 | 125 | 2000 | y = 1.70E-03x – 3.04E+00 | 0.990 |
| DP-C | 31.25-5000 | 6.25 | 31.30 | y = 9.88E-04x – 2.58E-03 | 0.998 |
| EP-N | 6.25-1000 | 1.25 | 6.25 | y = 1.52E-02x – 2.87E-01 | 0.996 |
| EP-C | 0.12-500 | 0.06 | 0.12 | y = 9.72E-02x + 6.05E-01 | 0.998 |
| FP-C | 6.25-1000 | 1.25 | 6.25 | y = 1.42E-02x + 1.15E-01 | 0.997 |
| GP-N | 500-5000 | 250 | 500 | y = 5.10E-03x – 2.23E+00 | 0.993 |
| GP-C | 250-10000 | 125 | 250 | y = 1.90E-03x – 1.83E-02 | 0.999 |

**Table S2** Precision and recovery of product peptides

| Peptide | % Recovery (n=6) | | | % Precision (n=6) | | | | |
| --- | --- | --- | --- | --- | --- | --- | --- | --- |
|  | LQC^a^ | MQC^b^ | HQC^c^ | LQC^a^ | | MQC^b^ | | HQC^c^ |
| AP-N | 90 | 89 | 95 | 9.8 | | 1.7 | | 2.8 |
| AP-C | 110 | 86 | 91 | 10.4 | | 1.9 | | 4.8 |
| BP-N | 87 | 121 | 112 | 3.6 | | 4.2 | | 2.4 |
| BP-C | 86 | 93 | 101 | 8.9 | | 2.1 | | 2.6 |
| CP-N | 88 | 101 | 96 | 13.9 | | 2.1 | | 3.7 |
| DP-N | 98 | 104 | 107 | 5.5 | | 5.7 | | 1.7 |
| DP-C | 112 | 110 | 113 | 13.2 | | 1.8 | | 2.4 |
| EP-N | 110 | 113 | 108 | 11.5 | | 2.0 | | 4.0 |
| EP-C | 113 | 109 | 105 | 5.6 | | 2.9 | | 1.4 |
| FP-C | 91 | 103 | 99 | 7.9 | | 3.0 | | 4.4 |
| GP-N | 86 | 100 | 108 | 5.2 | | 10.5 | | 7.7 |
| GP-C | 89 | 111 | 114 | 6.1 | 2.1 | | 3.8 | |

n, number of replicates.

LQC^a^, 2 or 2.5 times the LLOQ, as in Table 3.

MQC^b^, 0.5 times the highest concentration in the working range.

HQC^c^, 0.8 times the highest concentration in the working range.

## Supplementary Figures


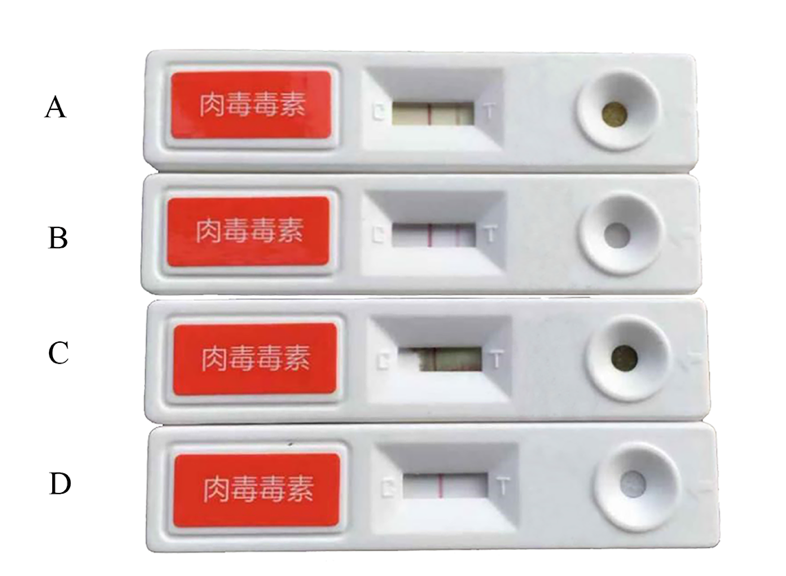


**Supplementary Figure 1.** LFA results for the vomitus samples of patients 1, 2 and 3 on day 1 after symptom onset. **(A)** The patient 1; **(B)** The patient 2; **(C)** The patient 3; **(D)** The blank control (PBS). Results showed the vomitus samples of the three patients were positive for BoNT, while without serotype differentiation.





**Supplementary Figure 2.** LC-MS/MS (MRM) peak areas ratios of product peptide and internal peptide versus product peptide concentration. Error bars were obtained from three parallel experiments. **(A)** AP-N. **(B)** AP-C. **(C)** BP-N. **(D)** BP-C. **(E)** DP-N. **(F)** DP-C. **(G)** EP-N. **(H)** EP-C. **(I)** GP-N. **(J)** GP-C. **(K)** CP-N. **(L)** FP-C

**
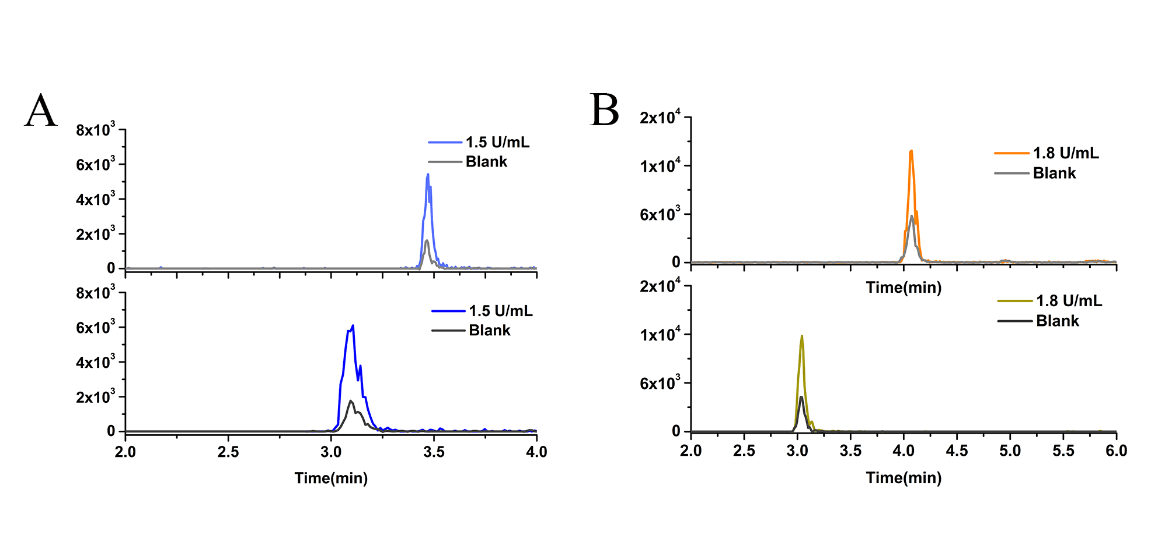
**

**Supplementary Figure 3.** LC-MS/MS (MRM) peak intensities for N-terminal products and C-terminal products in blank serum and serum spiked with BoNT/B or BoNT/E. **(A)** BP-N (upper lane) and BP-C (lower lane) obtained at blank serum and at LOD of BoNT/B (1.5 U/mL). **(B)** EP-N (upper lane) and EP-C (lower lane) obtained at blank serum and at LOD of BoNT/E (1.8 U/mL).


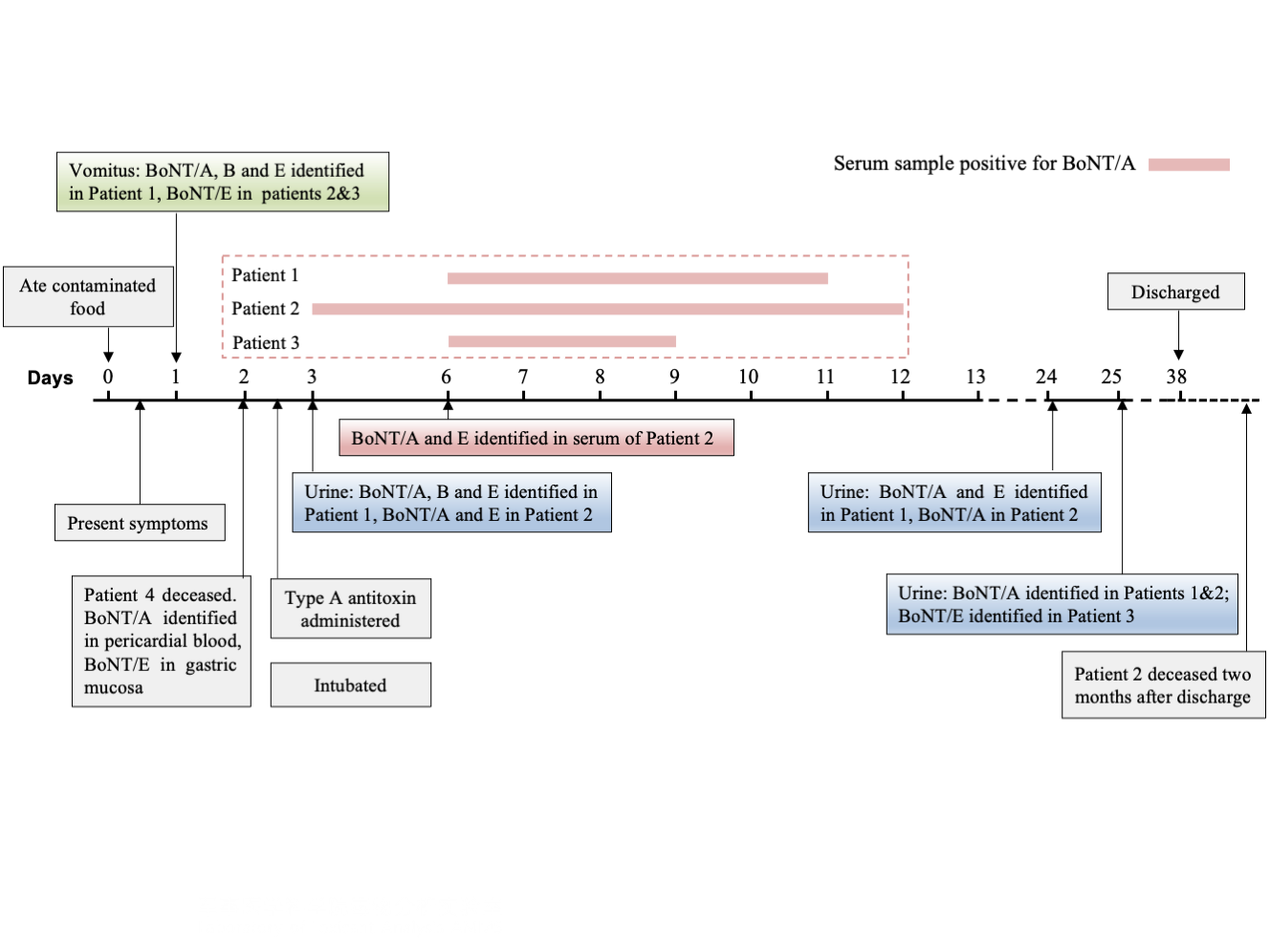


**Supplementary Figure 4.** Timeline of patients 1-4 diagnosed as mixed botulism caused by serotypes A, B and E
